# Lineage-resolved lifetime modelling reveals potential quiescence and synchronization among MDA-MB-468 breast cancer cells

**DOI:** 10.3389/fcell.2026.1911886

**Published:** 2026-09-14

**Authors:** Mónica Suárez Korsnes, Ellen Marie Botne Quinsgaard, Siver Andreas Moestue, Reinert Korsnes

**Affiliations:** 1 Department of Clinical and Molecular Medicine, Norwegian University of Science and Technology (NTNU), Trondheim, Norway; 2 Korsnes Biocomputing (KoBio), Trondheim, Norway; 3 Department of Pharmacy, Nord University, Bodø, Norway

**Keywords:** heritage, intermitotic time, lineage tracing, persister-like, single-cell tracking, sister cells, synchronisation

## Abstract

This study presents evidence of a previously unrecognised structured organisation of untreated cancer cell lineages and suggests an unexpected sensitivity to subtle experimental perturbations that is not readily explained by standard population models. These conclusions are based on exploratory analyses of cell-tracking data from untreated MDA-MB-468 breast cancer cells cultured under standard conditions. The analyses indicate that lineage-tracking data can reveal multiple layers of heterogeneity, synchronisation, and ancestry, together with stable quiescent, senescence-like, or persister-like states, even in the absence of external perturbation. These findings are hypothesis-generating rather than confirmatory and are intended to motivate future studies designed to test these hypotheses. The analysis is based on 72-h recordings from one or two wells in each of 21 independent experiments, comprising trajectories of 4,000 initial cells and their 34,152 descendants. During observation, 238 (6%) of the initial cells died, whereas 39 (1%) survived without dividing. The lifetimes of initial cells that died are well described by a conventional mixed gamma distribution, but the observed number of non-dividing survivors exceeds model predictions, suggesting a subpopulation with prolonged quiescent or senescence-like behaviour. The study further explores information extracted from healthy pedigree trees, defined by three consecutive normal cell divisions from the start of recording and no subsequent cell death. Only 22% of the 4,000 pedigree trees satisfy these criteria; the remainder exhibit abnormal divisions, prolonged intermitotic times, or cell death. Basic renewal theory indicates that initial cells tend to delay their first division, consistent with systematic differences between first- and second-generation intermitotic times. Finally, comparisons of pooled and experiment-specific lifetime distributions identify information-theoretic “elements of surprise”. Unexpectedly pronounced local maxima in empirical probability densities suggest high sensitivity to experimental conditions and may also reflect cellular synchronisation through intercellular communication together with ancestry-dependent inheritance.

## 1 Introduction

The field of single-cell biology has become a major area of biomedical research, as differences between individual cells and their behaviour are increasingly recognised to have a significant impact on cellular function (Symmons and Raj, 2016). Genetically identical cells can vary widely in gene expression, mRNA and protein abundance, as well as metabolic profiles (Elowitz et al., 2002; Cai et al., 2006; Zenobi, 2013; Bendall et al., 2014; Buettner et al., 2015; Deconinck et al., 2021). This variability is central to many biological and medical contexts, including cancer and immunology, where clonal populations are often assumed to behave uniformly (Greaves and Maley, 2012).

One key driver of this heterogeneity is reversible cell-state transitions where epigenetic mechanisms generate substantial phenotypic diversity (Sharma et al., 2010; Shaffer et al., 2017; Rosano et al., 2024; Bürger et al., 2026). This non-genetic heterogeneity is particularly relevant in cancer, where resistant or rare subpopulations may arise through epigenetic reprogramming (Spencer et al., 2009; Gupta et al., 2011; Pisco and Huang, 2015; Symmons and Raj, 2016; Shaffer et al., 2017).

Cancer cells exhibit high phenotypic plasticity and often maintain stable subpopulations over many generations (Gupta et al., 2011). Lineage-tracing therefore helps to understand how this plasticity shapes population dynamics (Meacham and Morrison, 2013; Lawson et al., 2018; Stuart and Satija, 2019; Qin et al., 2023). Evidence suggests that the cell of origin can influence tumour subtype and aggressiveness (Nowell, 1976; Ding et al., 2021; Vinuesa-Pitarch et al., 2021). Characterizing clonal cancer cells through heritable traits can thus elucidate the genetic and phenotypic drivers of cancer progression, facilitating the identification of novel therapeutic targets.

Central to these lineage dynamics is the degree of similarity between closely related cells. When sister cells exhibit greater similarity to each other than to randomly selected cells, it suggests an internal heritable component, indicating a high degree of non-genetic inheritance (Koren et al., 2015; Korsnes and Korsnes, 2018; 2023; Qin et al., 2023; Quinsgaard et al., 2024; Laisné et al., 2025). Despite increasing evidence for such inheritance, rigorous testing of unperturbed control cells remains surprisingly rare.

This study therefore investigates whether such classifications hold consistently across experiments and whether specific inherited phenotypes predispose certain lineages to dominate. As datasets expand, we can more rigorously address how intercellular interactions shape phenotypic patterns. Implementing such analyses could provide critical insights, enhancing both the interpretability and clinical relevance of *in vitro* models.

A key motivation of the present work is to assess whether untreated clonal cancer cells already display sufficient phenotypic variation to enable informative drug testing assays without prior perturbation or selective pressure. If untreated cells spontaneously adopt distinct proliferative or quiescent states, these intrinsic subpopulations may provide useful models for studying variability in therapeutic response. Identifying such lineages through computationally supported behavioural analyses provides an important rationale for this study, since most studies lack a proper baseline because they ignore lineage-resolved dynamics in untreated cells.

To address these questions, we characterize the lineages of untreated MDA-MB-468 breast cancer cells over 72 h using single-cell tracking and lineage tracing. The data set consists of 21 independent experiments including 38.152 cells, tracked across 4,000 lineages.

During proliferation, cells can propagate transcriptional states, epigenetic marks, protein abundances, and other molecular components to their daughters, producing lineage-specific phenotypes that can persist for multiple generations (Spencer et al., 2013; Yang et al., 2017; Davies et al., 2023; Laisné et al., 2025). This continuity enables lineage-resolved phenotyping rather than isolated single-cell measurements.

The work begins by presenting the joint probability distribution of intermitotic times for first- and second-generation sister cells. Consistent with previous observations, sister cells exhibit positively correlated intermitotic times (Konigsberg and Feister, 1986; Chakrabarti et al., 2018; Hughes et al., 2022), suggesting a partial inheritance of states related to the cell-cycle. However, the empirical distributions differed substantially between experiments. One possible explanation is that intermitotic time is sensitive to environmental factors, although this seems less likely in our case because the cells were untreated and exposed to relatively uniform conditions. An alternative hypothesis is that subtle interactions or synchronizing effects may emerge during experiments, potentially influencing lineage dynamics and population-level behaviour. If such interactions occur, they could have important implications for the design and interpretation of *in vitro* assays.

The data for the present work also revealed that the variability in intermitotic times increases following the first two divisions. This suggests that cells may require an adaptation period to the recording environment. While a mixed gamma distribution models the lifespan of non-dividing cells that die during the 72-h period, a subset of surviving non-dividing cells deviated from this model.

A key finding of this study is the identification of a rare non-dividing subpopulation of 39 cells, observed across 21 experiments during the 72 h recording period. The difficulty in identifying such rare cellular states at baseline without prior drug selection has historically presented a ‘needle-in-a-haystack’ dilemma in cancer biology. Because these spontaneous, non-dividing variants are exceptionally rare, their distinct molecular and behavioural signatures are entirely obscured in traditional bulk assays by the overwhelming signal of the actively proliferating majority (Sharma et al., 2010; Gupta et al., 2011; Liau et al., 2017; Guler et al., 2017).

These outliers may be biologically and clinically relevant, as rare non-genetic subpopulations within clonal cancer cell lines, since they have been shown to contribute to the emergence of drug-tolerant states following therapeutic exposure (Bell and Gilan, 2020; Goyal et al., 2023). The existence of pre-existing therapy-resistant or therapy-tolerant subpopulations prior to treatment has motivated efforts towards early identification and individualized therapeutic strategies with the goal of improving prediction of treatment response (Vendramin et al., 2021).

The use of long-term single-cell tracking was therefore essential for detecting these rare quiescent cells, as traditional bulk assays inherently obscure such variants within population-averaged measurements. Importantly, observing these cells in untreated *in vitro* conditions, in the absence of therapeutic or micro-environmental stress, supports the possibility that non-dividing subpopulations or persister-like phenotypes may arise from pre-existing intrinsic cellular states rather than exclusively from induced stress responses (Shen et al., 2020).

## 2 Materials and methods

### 2.1 Cell culture

The breast cancer MDA-MB-468, a triple negative epithelial cell line with amplified expression of the Epidermal Growth Factor Receptor (EGFR), was used in the present experiments. The cells were cultured in DMEM:F12 with HEPES (Gibco, 3133038), supplemented with $50{UmL}^{-1}$ penicillin-streptomycin (P/S) (Gibco, 15070063), and 5% foetal calf serum (FCS) (Gibco, 10270).

### 2.2 Data set collection

The dataset for the present study is derived from 21 independent experiments originally conducted for two independent projects with objectives different from those of the present study. The first 17 experiments were conducted as part of the study by Quinsgaard et al. (2024), whereas the remaining four experiments were carried out as part of the master’s thesis by Wardy (2025). To ensure consistency, the analysis was restricted to cells maintained under standard basal culture conditions without exposure to experimental treatments. Although the video data were obtained from baseline control wells on plates that also contained treated experimental groups, the cells analysed in these wells remained entirely untreated. These baseline control data therefore satisfy the inclusion criteria for the present study.

### 2.3 Microscopy

Time-lapse recordings of the first 17 experiments were acquired using a Zeiss TIRF III fluorescence microscope. The cells were kept at 37 °C in a humidified 5% C $O_{2}$ atmosphere. The recordings were recorded with 10× magnification in phase-contrast mode, with images captured every 2.5 min for a total duration of 72 h. For each microscopy experiment, cells were seeded at a concentration of 200cells/mm 2 in 96-well culture plates with microscopy-grade µClear polymer bottoms (Greiner Bio-One, 655090). From each well, two fields of view with dimensions 1.33×1.33 mm^2^ were recorded.

The second time-lapse recordings of the remaining four experiments were captured using the Nikon Crest v3 Spinning Disk Confocal Microscope, equipped with dual Kinetix sCMOS cameras (2,720 × 2,720 pixels). To ensure physiological relevance, cells were maintained in a controlled environment using the integrated Tokai hit Warming Box incubation system, set to 5% C $O_{2}$ atmosphere at 37 °C under humidified conditions. The imaging was conducted in differential interference contrast (DIC) mode using a ×10 magnification objective, with images captured every 3 min for a total duration of 72 h. Imaging fields of view were carefully centred in each well to optimise both cell density and trackability. Focus was maintained over the 72 h imaging period using a Perfect Focus System (PFS, Nikon) hardware autofocus, supplemented by manual adjustments as necessary.

Given the extended duration of image acquisition, a label-free imaging approach was used to enable continuous monitoring while minimising experimental perturbation. Accordingly, Differential Interference Contrast (DIC) microscopy was selected because it avoids photobleaching and phototoxicity associated with fluorescence-based techniques, thereby facilitating long-term observation of dynamic cellular processes while preserving cell viability and physiological function.

### 2.4 Time-lapse video microscopy

Video tracking of the MDA-MB-468 breast cancer cell line was used to monitor individual cell behaviour and trace lineage relationships. 21 independent experiments were conducted, each involving 72-h recordings of untreated control cells cultured in test plates under undisturbed conditions. This gave 76 recordings of cells cultured under comparable conditions. In total, over 38,152 control cells were tracked.

### 2.5 Cell tracking

Korsnes Biocomputing (KoBio)^1^ conducted the actual video tracking of individual cells from the microscopy data using its specialised Kobio Celltrack software which in principle could be replaced by similar systems (Tinevez et al., 2017; Padovani et al., 2022; Aragaki et al., 2022) The initial stage involved localising all cells within a central rectangular region of the video frame at the start of the recording. This rectangle was defined with fixed margins relative to the edges of the field of view, precisely large enough to include 50 or 100 cells. Subsequently, the positions of these cells, as well as their progeny, were continuously monitored throughout the 72-h recording period. The identification of sisters during the second division is visualised using the KoBio cell tracking system (Figure 1).

**FIGURE 1:**
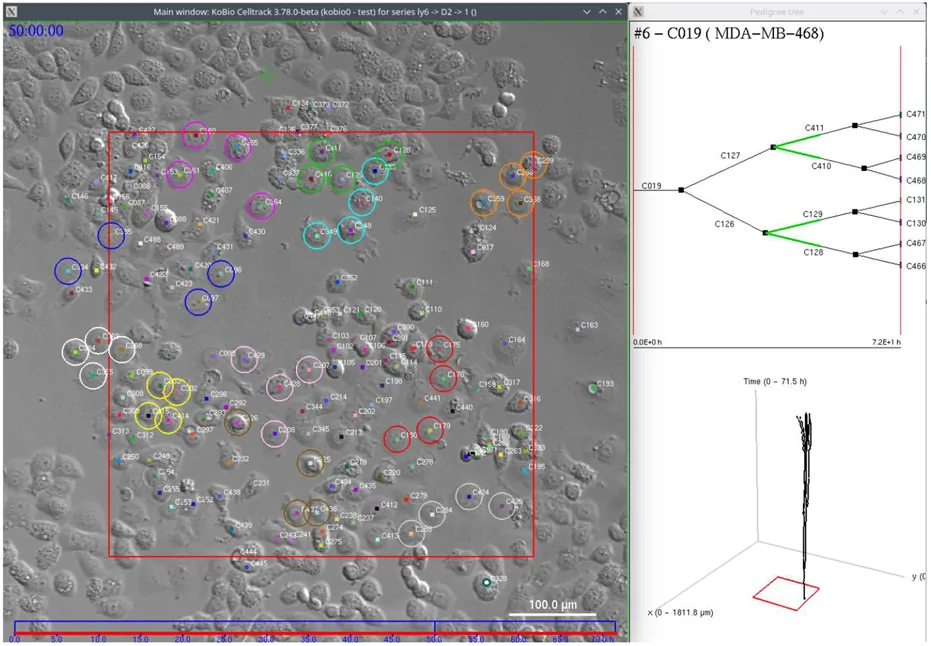
Illustration showing the second generation of sister cells. Each coloured circle represents two pairs of sister cells belonging to the same lineage tree.

The initial tracking phase generates raw positional coordinates that contain noise and identification ambiguities, potentially biassing subsequent calculations of track length and migration speed. To reduce these errors, Gaussian kernel smoothing (Wand and Jones, 1994) regularised the data (Korsnes and Korsnes, 2023) using a fixed bandwidth of 5 min. The programme system is also used for observer-guided registration of cell death and its classification into the two broad categories of necrosis and apoptosis. It is also used to collect data on multipolar cell division and cell fusion and re-fusion events (Korsnes and Korsnes, 2017).

Figure 2 shows an example of a cell that is observed not to divide throughout the entire 72-h recording. The cell exhibits a limited period of movement, which may reflect crucial events subject to further investigation.

**FIGURE 2:**
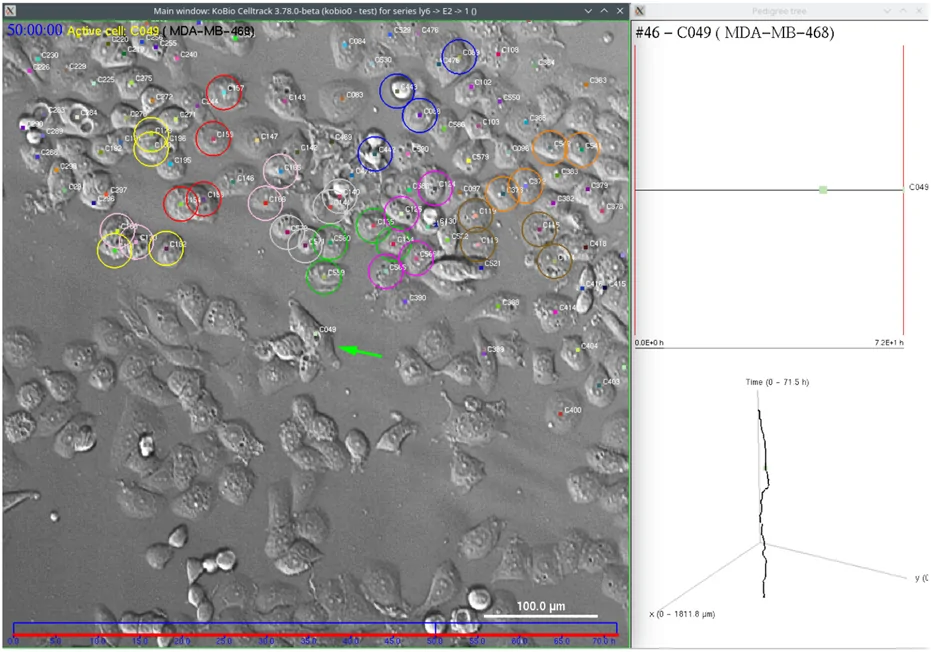
Illustration showing a cell (green arrow) that did not divide during the 72 h recording period. Note the last row of experiment Ly6, summarized in Table 1. This outlier (with ID C049) was observed throughout the entire recording period. Each coloured circle represents two pairs of second-generation sister cells belonging to the same lineage tree.

## 3 Results

### 3.1 Viable and quiescent cells

This work focuses on a specific class, Ω , of “healthy” cells, defined as those belonging to pedigree trees spanning complete generations I and II, with no subsequent cell death observed. Figure 3 illustrates a lineage of such “healthy” cells, beginning with the root cell C016.

**FIGURE 3:**
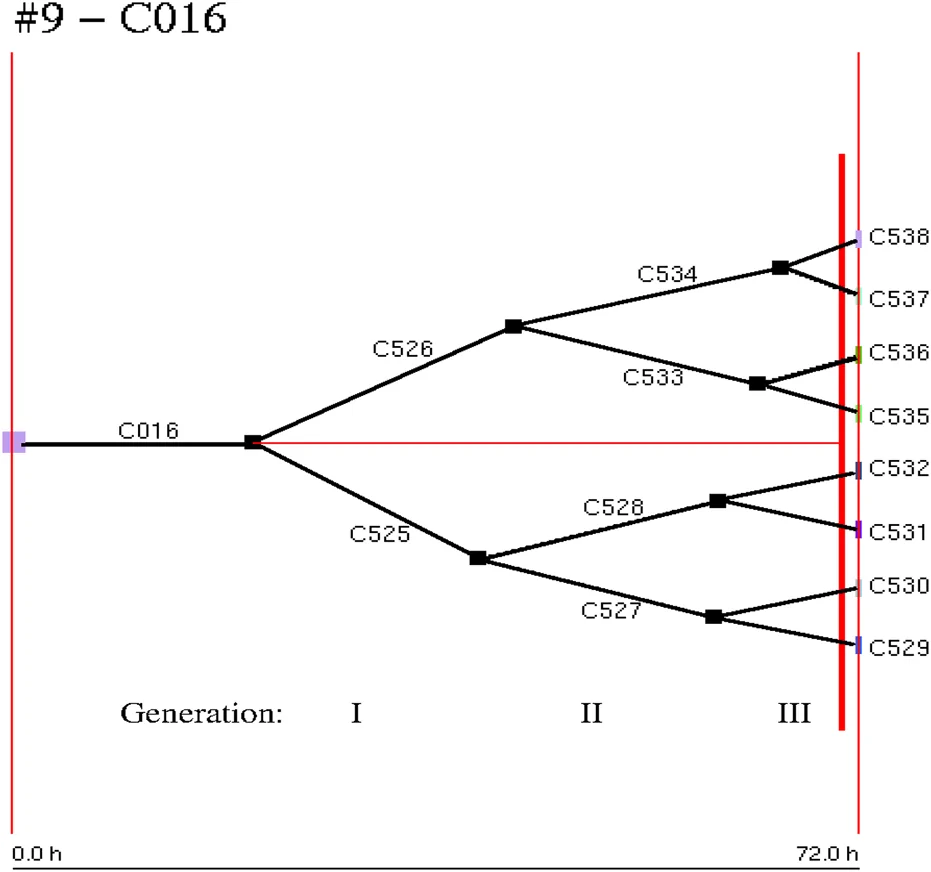
Pedigree tree comprising cells of class “healthy”, Ω , spanning complete life cycles of generations I, II, and III, with no observed cell death. The tree contains 15 cells labelled with their IDs. The bold vertical red line represents 60 h after the first cell division (of C016, which is also called *root cell* or *initial cell*), serving as a possible baseline for consistent comparison between different pedigree trees.

The present work performs analyses restricted to such highly viable cells. Approximately 22% of the current cell lineages meet this criterion (out of a total of 4,000 pedigree trees). The end state of the 38,152 cells is summarised in Table 2. Note that among mitotic events, approximately (1.32%) can undergo abnormal cell divisions.

Furthermore, 39 initial cells (1%) survived throughout the entire 72 h recording period without dividing, while 238 initial cells died before division. Table 1 summarises the observations of the former set of cells.

**TABLE 1:** Summary of frame counts for 39 non-dividing initial cells across wells and experiments that survived throughout the 72-h recording period. These data were obtained by tracking 4,000 initial cell lineages, of which approximately 1% survived the recording period without undergoing cell division. The 12 experiments KT8–KT27 each included two wells, with two disjoint frames per well, each initially containing 50 tracked cells, giving 2,400 pedigree trees. The experiments KT30 and KT31 each included three wells, giving 600 pedigree trees. The three experiments siKT4–siKT6 each included one well with two frames and 100 initial cells, giving 600 pedigree trees. The four experiments Ly2–Ly6 each included one well with a single frame initially containing 100 cells, giving 400 pedigree trees.

Experiment	# Cells	Well 1		Well 2		Well 3		Sum
		1	2	3	4	5	6	
KT8	200	​	1	​	​	​	​	1
KT12	200	​	​	​	​	​	​	​
KT13	200	2	4	1	1	​	​	8
KT14	200	​	​	​	​	​	​	​
KT15	200	​	​	1	1	​	​	2
KT16	200	​	​	​	2	​	​	2
KT18	200	​	​	​	​	​	​	​
KT19	200	2	​	1	​	​	​	3
KT23	200	​	​	2	​	​	​	2
KT24	200	​	2	5	​	​	​	7
KT25	200	3	​	1	​	​	​	4
KT27	200	1	​	1	​	​	​	2
KT30	300	​	​	2	​	1	​	3
KT31	300	​	​	​	​	​	2	2
siKT4	200	​	​	​	​	​	​	​
siKT5	200	​	​	​	​	​	​	​
siKT6	200	​	1	​	​	​	​	1
Ly2	100	​	​	​	​	​	​	​
Ly3	100	​	1	​	​	​	​	1
Ly4	100	​	​	​	​	​	​	​
Ly6	100	​	1	​	​	​	​	1

This work restricts statistical analyses to particularly viable cells. Approximately 22% of the current cancer cell lineages meet this criterion (out of a total of 4,000 pedigree trees). Furthermore, initial 39 cells (1%) survived throughout the entire 72 h recording period without dividing, while 238 initial cells died before division as shown Table 1.

Figure 4 shows a histogram of the lifetime probability density for the 238 non-dividing cells that died during the recording.

**FIGURE 4:**
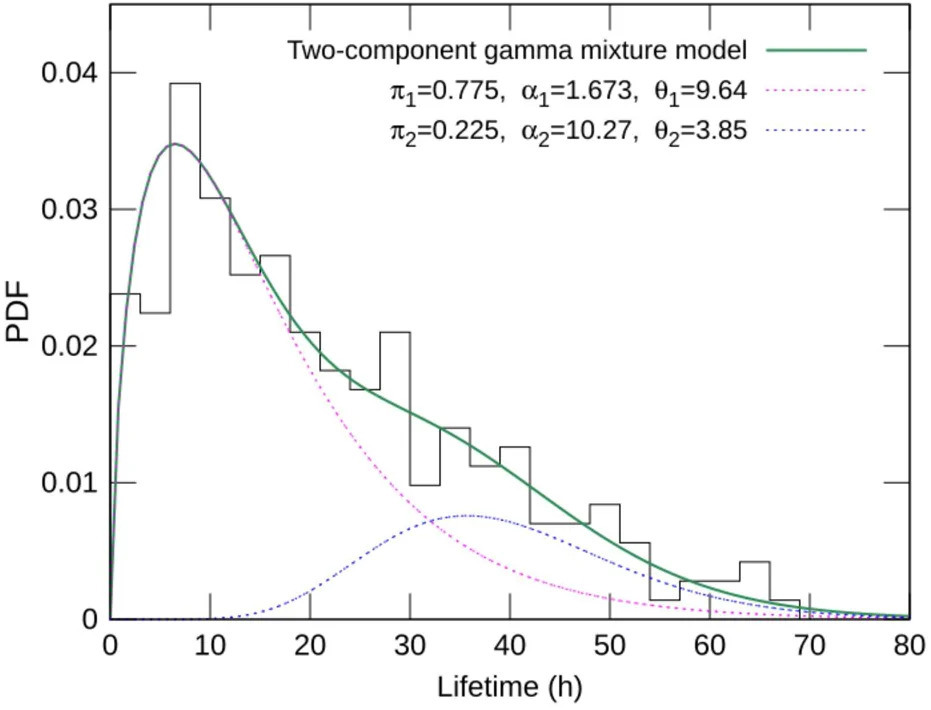
Histogram of lifetimes for the 238 cells that died without division during the 72 h recording period (out of 4,000 initial cells). These data were fitted with a two-component mixture of gamma distributions having mean lifetimes of 16.1h and 39.5 h and corresponding weights $\pi_{1}=0.775$ and $\pi_{2}=0.225$ . The fitted mortality model suggests that the distribution of lifetimes is effectively confined to the 72 h observation period, with less than 0.5% of the probability mass beyond this time. However, 39 of the 4,000 initial cells survived until the end of the recording period. These cells constitute a distinct subpopulation whose observed survival proportion is substantially greater than predicted by the fitted mortality model (39/277≈14%) , suggesting that this subset is not adequately described by the fitted lifetime distribution. This observation supports the hypothesis that a fraction of cells may exhibit prolonged quiescence or a senescence-like state.

The figure also includes a fit of a two-component Gamma mixture distribution to these data (Lakshmi and Vaidyanathan, 2016):

$$g\left(x\right)=\pi_{1}\;f\left(x;\alpha_{1},\theta_{1}\right)+\pi_{2}\;f\left(x;\alpha_{2},\theta_{2}\right)$$ (1)

where $\pi_{1}+\pi_{2}=1$ and

$$f\left(x;\alpha ,\theta \right)=\frac{x^{\alpha -1}\exp\left(-x/\theta \right)}{\theta^{\alpha }\Gamma \left(\alpha \right)}.$$ (2)

The Gamma distribution (Equations 1, 2) is a generalisation of the exponential distribution, and it typically provides a good model for lifetime distributions of complex systems. It appears to fit the data in Figure 4, yielding, for example, a Kolmogorov–Smirnov (KS) test p -value greater than 0.9 (see the supplementary data). However, caution must be exercised not to over-interpret this observation, since the model parameters were estimated from the same data used to assess the fit. Consequently, the result should be regarded as descriptive rather than confirmatory. Nevertheless, it may serve as a useful hypothesis-generating observation within this exploratory data analysis.

Note that the model fit in Figure 4 is effectively confined to lifetimes below 72h, with negligible probability mass beyond this threshold. In an approximate Hilbert-space interpretation, the distribution of lifetimes of cells that survive longer than 72 h can therefore be regarded as orthogonal to the fitted distribution. Consequently, a least-squares fit to the data below 72 h is effectively independent of the distribution of lifetimes beyond this threshold.

The present single cell tracking demonstrates that, among the current 4,000 initial cells, a fraction of the 39 non-dividing survivors may withstand treatment by evading vulnerabilities specifically associated with active cell division. If they can reverse their quiescent state, this phenomenon may be of medical interest, particularly in relation to long-term clinical risk (Cai et al., 2006; Buettner et al., 2015; Cornwell et al., 2023; Davies et al., 2023).

### 3.2 Delay of first cell division

Cells at the beginning of the present video recordings tend to exhibit delayed division. Renewal theory (Cox, 1962) helps to reveal this delay, assuming that the cells had grown under stable conditions over long time scales before the transition to time-lapse microscopy. Synchronisation among cells can, however, affect estimates of the interval lifetime or the forward recurrence time distributions. There is no particular reason to believe that this synchronisation is a consistent side effect of the present experimental protocol. Cells were simply transferred from routine culture and seeded in wells 24 h before recording.

Assuming the equilibrium described above and that the intermitotic time, τ , is constant and equal to $\tau_{\text{c}}$ , the waiting time, W , until an initial cell divides is uniformly distributed throughout the interval $\left[0,\tau_{\text{c}}\right]$ . Consequently, its probability density function (PDF) is

$$f_{W}\left(t\right)=\left\{\begin{array}{cc} 1/\tau_{\text{c}} & \text{if }0\leq t\leq \tau_{\text{c}},\\ 0 & \text{otherwise.} \end{array}\right.$$ (3)

This is easily understood by viewing the start of the recording as a random point in time. Renewal theory provides a more general expression for the probability density function:

$$f_{W}\left(t\right)=\frac{1}{E\left(\tau \right)}\left(1-F_{\tau }\left(t\right)\right)$$ (4)

where $F_{\tau }\left(t\right)$ denotes the cumulative distribution function of τ . Note that $F_{\tau }\left(t\right)=0$ for t below the minimum intermitotic time. Therefore, $f_{W}\left(t\right)=1/E\left(\tau \right)$ is constant over this initial interval.

A key assumption underlying the model Equations 3, 4 is that time-lapse microscopy and experimental handling of cells immediately prior to recording have no influence on their subsequent division. Figure 5 contradicts this assumption, showing a discrepancy between the empirical and model distributions.

**FIGURE 5:**
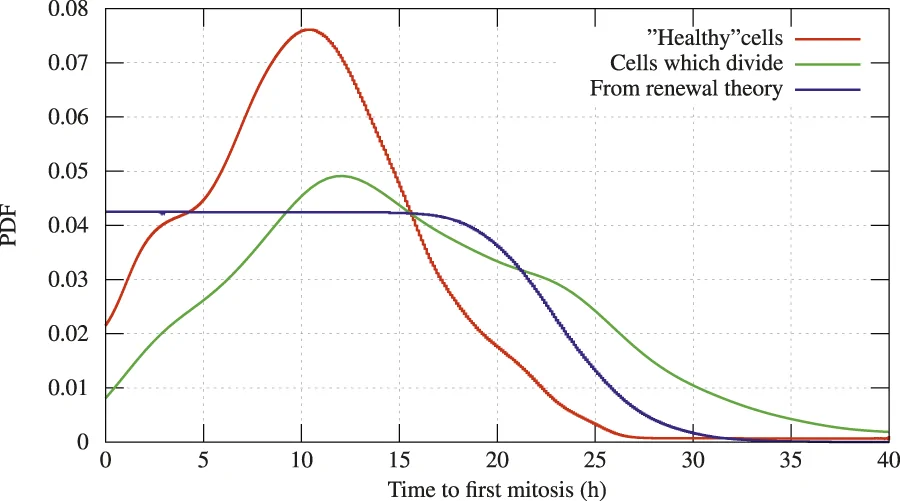
Distribution of the waiting time, W , from start of recording until an initial cell divides (Gaussian kernel density estimates). Red: “Healthy” cells, Ω . Green: Cells dividing within 72 h. Blue: Model distribution based on lifetimes of generation I among “healthy” cells (see text). The discrepancy between the empirical and model distributions suggests that cells need time to adapt to the recording conditions. Handling prior to recording may also influence cell division during the initial part of the recording.

Here, the expectation E(τ) and the cumulative distribution function $F_{\tau }$ are pragmatically estimated from the lifetimes of generation I “healthy” cells, Ω .

The figure illustrates how environmental factors can affect cell division. This influence may be regarded as an unwanted experimental artefact. However, such observations may also stimulate ideas for influencing or regulating cell division in the context of cancer treatment.

### 3.3 Variability among experiments

Figure 6 presents statistical relations between the intermitotic time, τ , for generation I and II sister cells classified as “healthy”, Ω , aggregated across all 21 experiments.

**FIGURE 6:**
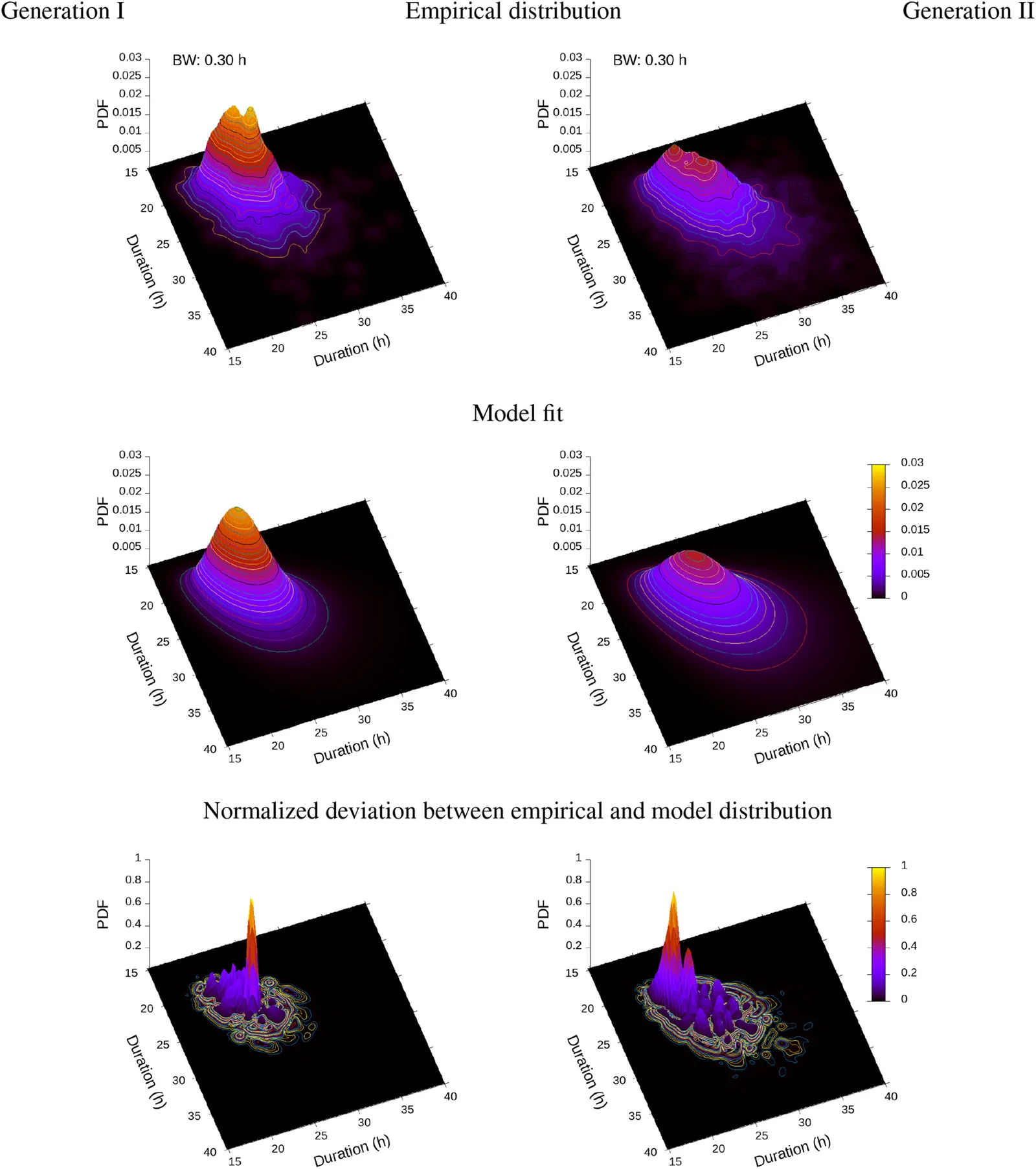
Summary statistics for the intermitotic lifetimes, τ , of all “healthy” sister cells, Ω , from 21 experiments. Left: Generation I. Right: Generation II. Upper row: Empirical joint distribution obtained by Gaussian kernel smoothing with a bandwidth BW=0.3 h . Note the positive correlation between life-lengths of sister cells indicating forms of inheritance. Korsnes et al. (2026) illustrates the effect of the bandwidth for generation I and II. Middle row: Fitted bivariate log-log-normal distribution. Lower row: Normalized deviation between the fitted model and the empirical distribution. Two distinct lifetime classes appear to emerge in generation II, with density maxima at approximately 24 h and 29h, consistent with the observations in Figure 7.

The upper row presents the empirical joint distributions for these generations in the form of the Gaussian kernel density estimate with bandwidth 2.5 h. It shows an accumulation of divisions around two close maxima around 24 h. Generation I seems to exhibit a higher degree of correlation compared to generation II.

The middle row shows the results of fitting a simple log-normal model distribution to the data. Meanwhile, the lower row displays a normalised measure of the deviation between the empirical and model distributions. This deviation is defined as follows:

$$f\left(x,y\right)\overset{\text{def}}{=}|m\left(x,y\right)-p\left(x,y\right)|\;p\left(x,y\right)m\left(x,y\right)$$ (5)

where p(x,y) and m(x,y) represent the empirical and model joint distributions, respectively. This approach aims to visualise the “element of surprise” (entropy) in the data by highlighting deviations from the simplified general average. In particular, the distribution of generation II appears to exhibit two main modes of intermitotic time.

Figure 7 shows the distribution of intermitotic times for two subsets of these data. One subset comprises cells with sister cells that divide within 1.5 h of each other (“similar sisters”), while the other consists of cells with sister divisions separated by more than 2.5 h (“different sisters”). Consequently, generation II exhibits a more distinct bimodal distribution with a consistent similarity between the two groups.

**FIGURE 7:**
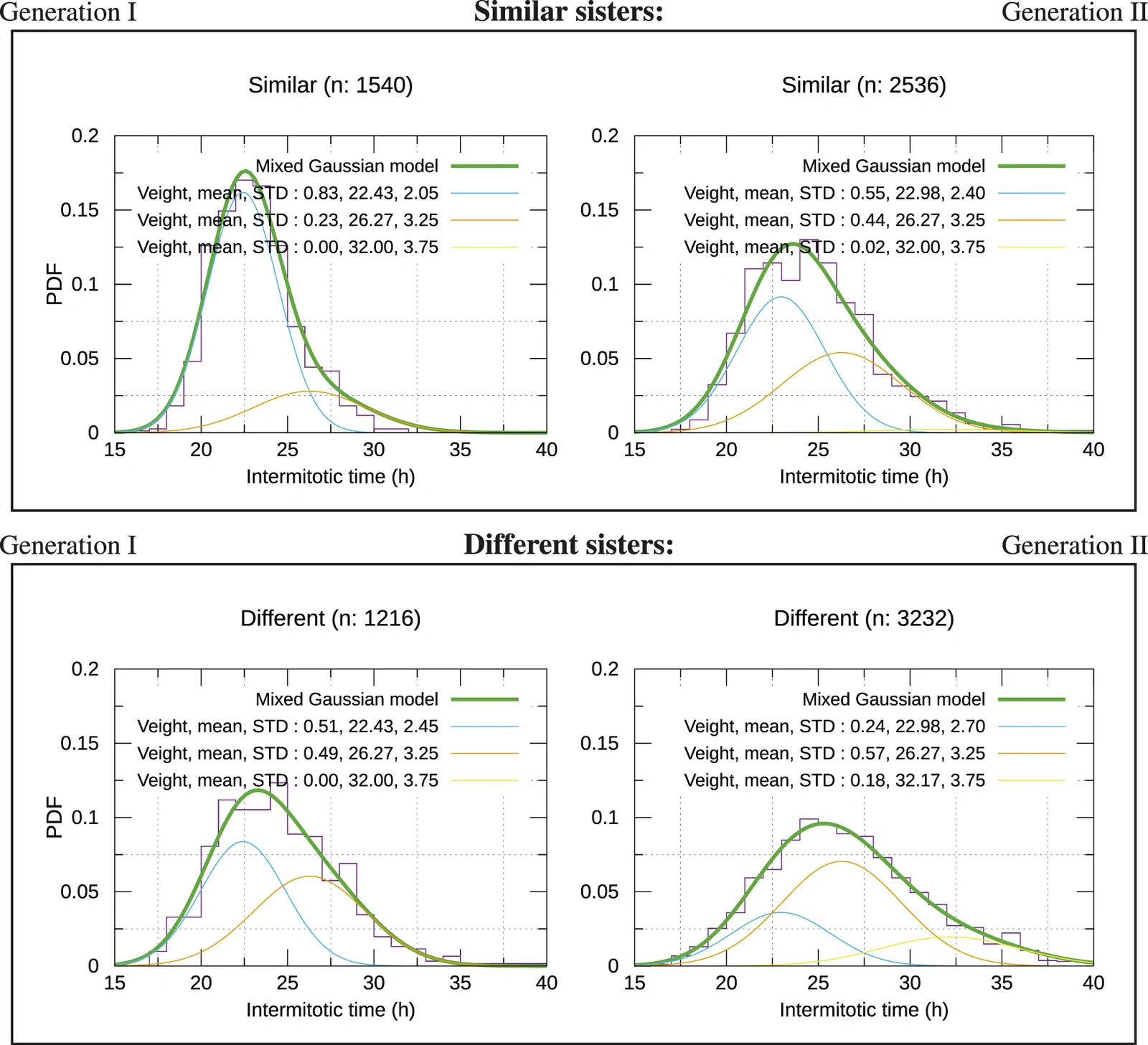
Simple Gaussian mixture modelling of intermitotic lifetimes, τ , for generation I (left) and II (right) sister cells classified as “healthy”, Ω . Upper panel: Distribution of intermitotic times for cells whose sister cell divides within 1.5 h of their own division. Lower panel: Distribution of intermitotic times for cells whose sister cell divides more than 2.5 h apart. Despite its simplicity, the model suggests the presence of two dominant “modes”, with mean intermitotic times of approximately 22.5 and 26.3 h.

Figures 8–11 show the corresponding “element of surprise” according to Equation 5 for each experiment considered separately. Equations 6–11 represents and early attempt to quantify this “surprize” of observations. The results indicate that the distribution of the lifespans of the sister-cells differs from the aggregated distribution (Figure 6), suggesting that the intermitotic time may serve as a proxy for more general experimental-specific cellular behaviour.

**FIGURE 8:**
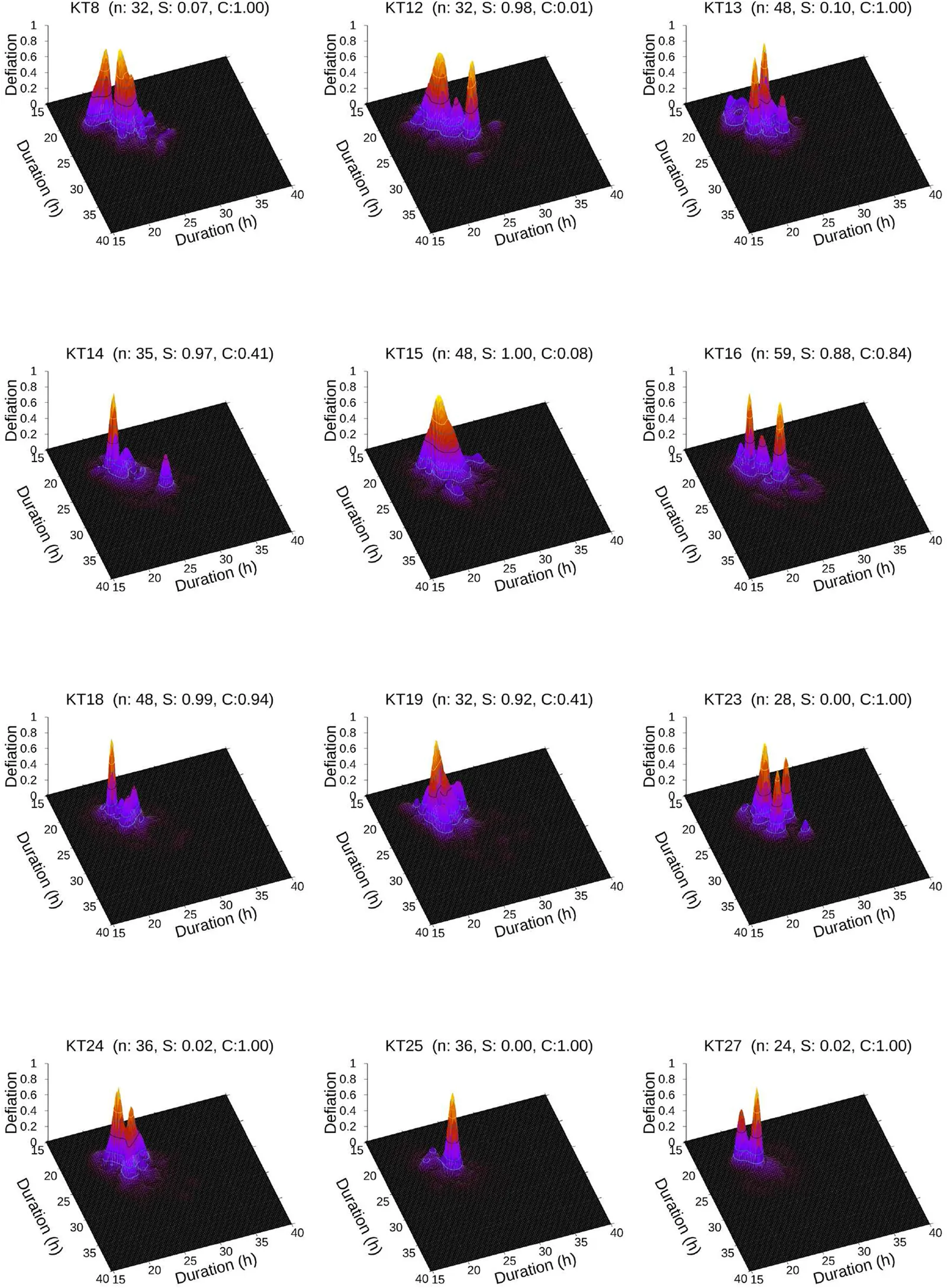
Surprise of pairwise sister-cell intermitotic times (*cf.* Equation 5), τ , for generation I “healthy” cells, Ω , across 21 experiments. See text.

**FIGURE 9:**
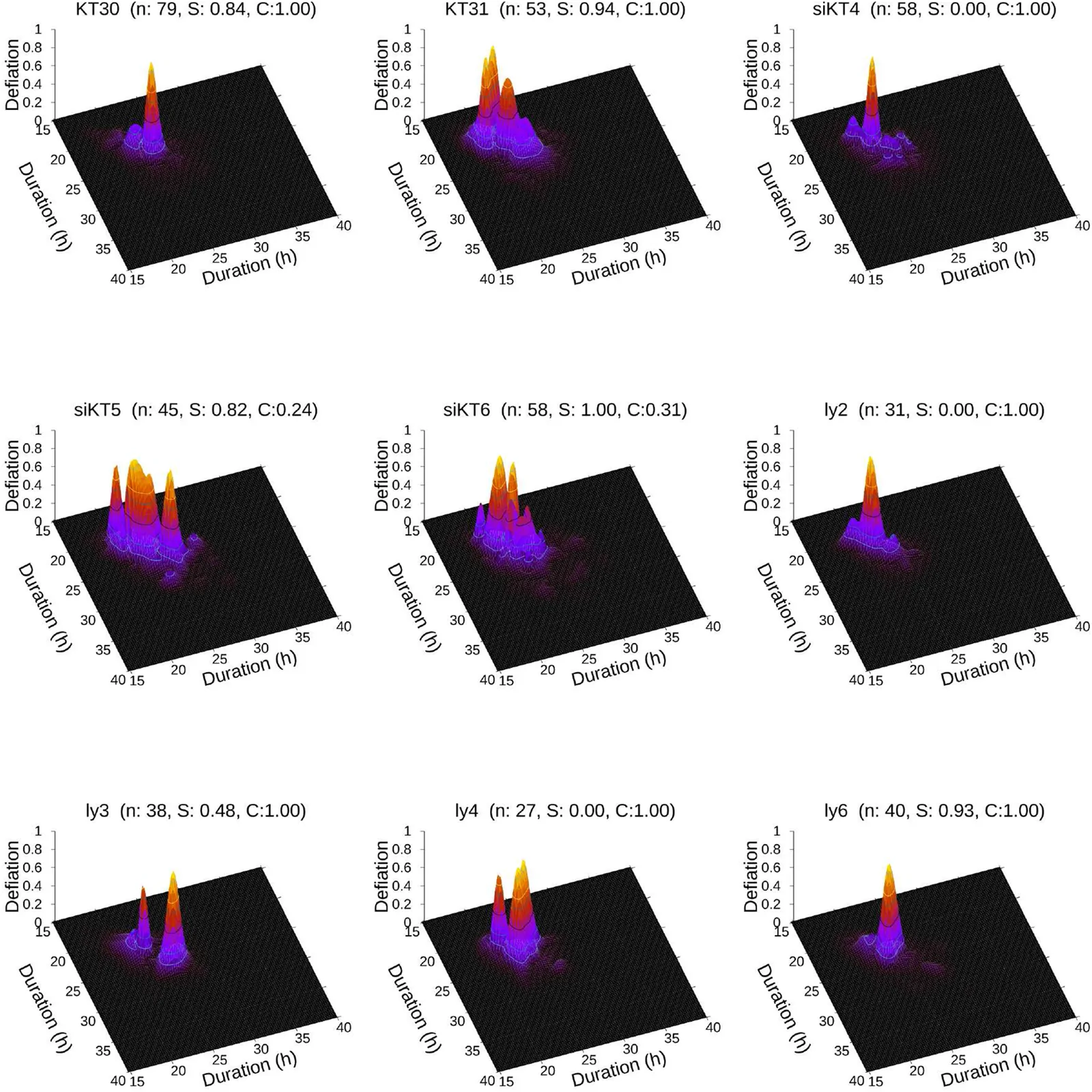
Surprise of pairwise sister-cell intermitotic times (*cf.* Equation 5), τ , for generation I “healthy” cells, Ω , across 21 experiments. See text.

**FIGURE 10:**
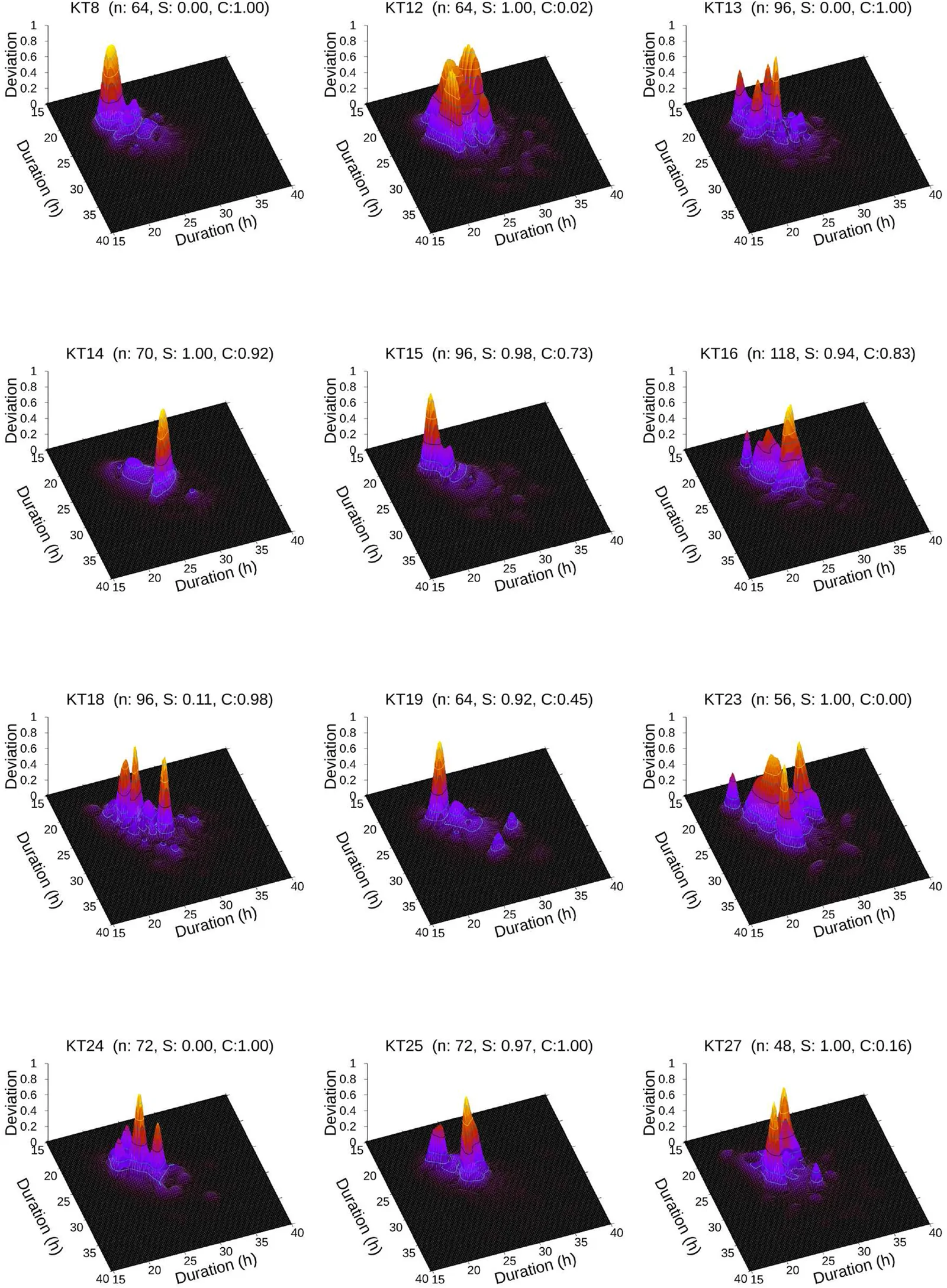
Surprise of pairwise sister-cell intermitotic times (*cf.* Equation 5), τ , for generation II “healthy” cells, Ω , across 21 experiments. See text.

**FIGURE 11:**
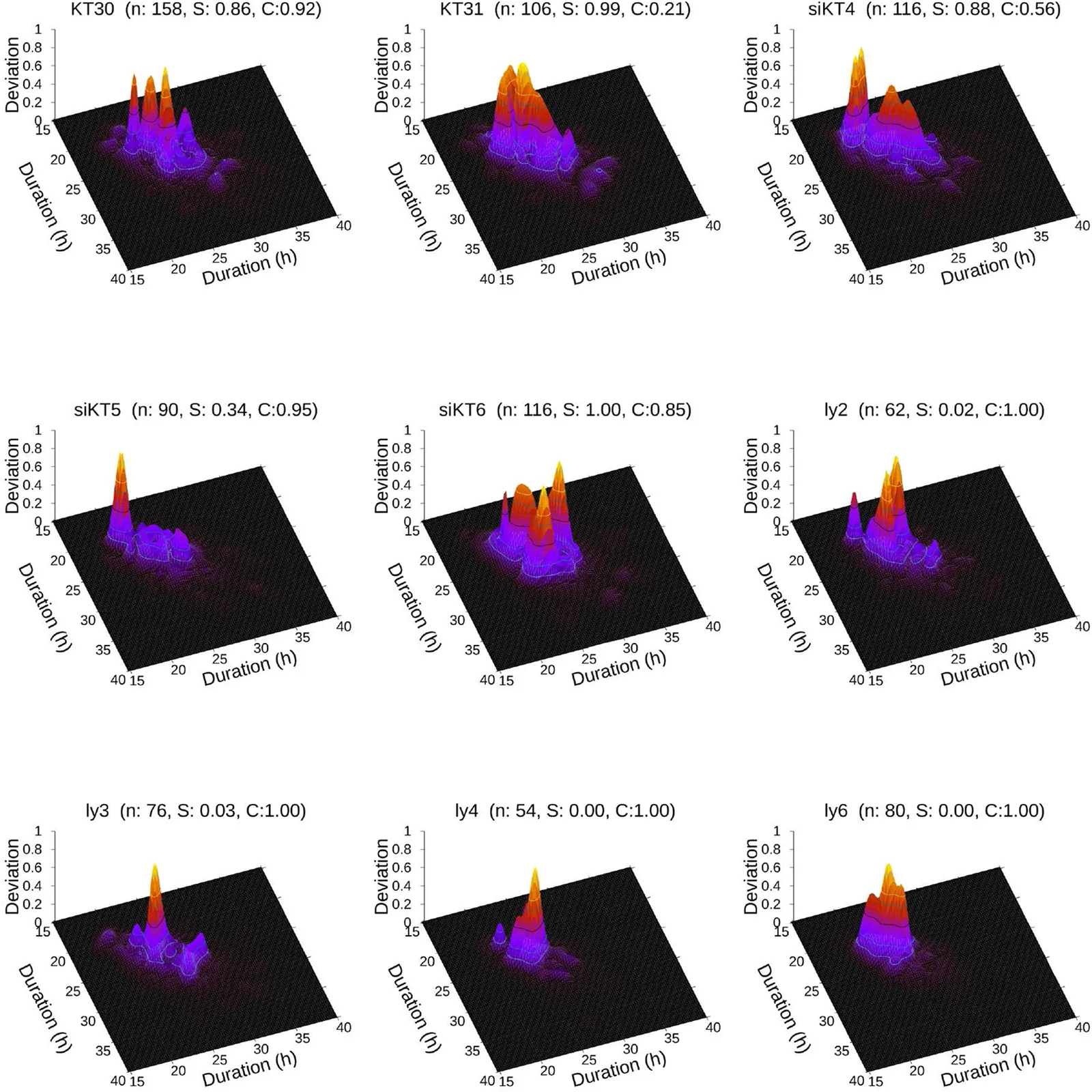
Surprise of pairwise sister-cell intermitotic times (*cf.* Equation 5), τ , for generation II “healthy” cells, Ω , across 21 experiments. See text.

Assume that the two-component vectors $X_{i}$ , i=1,2,…,n , represent observed lifetimes for n pairs of sister cells in an experiment E . Let p(X) denote the overall model probability density of the two-component stochastic variable X (Figure 6). The formula

$$I_{E}=-\underset{i}{\sum }\log\left(p\left(X_{i}\right)\right)$$ (6)

is a common measure of information (Shannon, 1948) in these data. A large value signifies a high degree of “surprise” (or information) in the observations.

Furthermore, let $Y_{i}$ , i=1,2,…,n , denote hypothetical (simulated) measurements drawn according to the overall model distribution p . Then

$$I_{H}=-\underset{i}{\sum }\log\left(p\left(Y_{i}\right)\right)$$ (7)

provides, similarly to Equation 6, a measure of the surprise associated with a simulated dataset.

The quantity

$$S=P\left(I_{E}>I_{H}\right)$$ (8)

represents the fraction of simulations for which the information (surprise) of the observed data exceeds that of the simulated data. Figures 8–11 provide values of S .

Figures 8–11 also provide a simple measure, C , of the “clustering” of data, which is inspired by mechanical physics. Assume, as above, that the two-component vectors $X_{i}$ , i=1,2,…,n , represent observed lifetimes for n pairs of sister cells in an experiment E , and that $Y_{i}$ , i=1,2,…,n , denote hypothetical (simulated) measurements based on the overall model distribution.

The formula

$$U_{E}=-\underset{i}{\sum }\underset{k\neq i}{\sum }\frac{1}{\left|X_{k}-X_{i}\right|}$$ (9)

resembles the gravitational potential energy for the set of positions $X_{i}$ . A similar formula applies for data from simulation H :

$$U_{H}=-\underset{i}{\sum }\underset{k\neq i}{\sum }\frac{1}{\left|Y_{k}-Y_{i}\right|}$$ (10)

If the “particles” clump together as they would in a gravitational field, the gravitational potential energy decreases. Figures 8–11 provide the “clustering index”

$$C=P\left(U_{E}<U_{H}\right)$$ (11)

which represents the fraction of simulations where the potential energy is lower for the observed data compared to the simulated data.

## 4 Discussion

Evaluating cell behaviour under baseline conditions is crucial for understanding the intrinsic capacity of cell populations to generate non-genetic heterogeneity and variability between experiments. Traditionally, *in vitro* assays have followed a “drug-first” paradigm, in which cell populations are analysed only after exposure to therapeutic agents. The responses are then compared with the untreated control cells, which are often implicitly assumed to represent a homogeneous or “standard” baseline that is insensitive to small perturbations. This approach introduces a significant confounding factor because it can be difficult to distinguish behaviours induced by drug stress from those arising from pre-existing intrinsic cellular states (Hata et al., 2016; Goyal et al., 2023; Russo et al., 2024). The present study aims to overcome this limitation by motivating the systematic characterisation of untreated cell populations. This approach allows pre-existing heterogeneity to be studied directly, rather than inferred indirectly from responses to therapeutic perturbations.

The generality and simplicity of the present approach may pave the way for automated, standardised analyses of untreated control cells in the context of *in vitro* assays. The study should be straightforward to reproduce. It is based on tracking, a widely available cell line maintained under standard culture conditions. The primary observable is inter-mitotic time, a relatively objective measurement that serves as a proxy for the complex and sensitive biological processes governing cellular proliferation. Future microscope systems could integrate these standardised analyses directly into their acquisition or analysis software, enabling real-time monitoring and early detection of unexpected cellular behaviours. Such capabilities could be valuable for both basic research and biomedical applications.

We analyse 4,000 unperturbed MDA-MB-468 cell lineages and observe extensive phenotypic heterogeneity at the single-cell level. Only 22% of the initial cells generated “healthy” pedigree trees, defined as lineages that underwent at least three consecutive divisions without cell death. This unexpectedly low fraction suggests that sustained proliferative expansion is not the predominant outcome, even in the absence of therapeutic pressure. The remaining 78% of lineages fell outside these criteria, displayed various aberrant outcomes, including refusions, multipolar divisions, prolonged intermitotic times, and cell death (Table 2). These observations challenge the common assumption that established cancer cell lines maintained under standard conditions predominantly undergo stable proliferative expansion.

**TABLE 2:** Summary of the final cell state for the observed 38,152 cells tracked during the 72-h recording period. A fraction of 1.3% of the cell divisions are multi-polar. About 3.5% of the cells died through apoptosis causing debris to be engulfed by the others.

Cell state	Live	Mitosis	Refusion	Fusion	Multipolar divisions	Apop	Nec	Out of scene	Total
Count	18364	16649	1,017	295	224	1,345	257	1	38152
%	48	44	2.7	0.77	0.59	3.5	0.67	0.003	100

Non-genetic heterogeneity and sensitivity to small perturbations appear to be intrinsic features of baseline population dynamics, prior to therapeutic intervention, and may therefore contribute to variability in subsequent treatment responses. One possible source of such sensitivity is synchronisation or flocking behaviour within the cell population. Situations exhibiting this type of sensitivity cannot always be meaningfully counteracted through improved experimental control, but instead require statistical characterisation. The situation is analogous to throwing dice: individual outcomes cannot be predicted deterministically, whereas their statistical properties can be characterised with high precision.

A total of 238 initial cells died during the 72-h recording period, and their lifetimes are well described by a common survival model that predicts that virtually no cells should survive beyond the recording period (Figure 4). However, lineage tracking identified a distinct subpopulation of 39 cells (1%) that remained non-dividing throughout the entire observation period. These cells fall well outside the range predicted by the fitted survival model and therefore cannot be regarded as the long-lived tail of the same lifetime distribution. Instead, they constitute a statistically distinct component of the population. Unlike the transient delays in cell-cycle progression observed in other lineages, these cells exhibited persistent proliferative arrest throughout the duration of the experiment. This behaviour is consistent with a stable non-proliferative state and may reflect quiescent or senescence-like phenotypes within the untreated population. It may also reflect a persister-like state in which cells exhibit a pre-existing slow proliferative rate. These cells may exist before drug treatment and are then selected by treatment, following classical Darwinian rules (Shaffer et al., 2017; Shen et al., 2020).

Notably, the emergence of such cells in the absence of external perturbation suggests that substantial phenotypic stratification may already exist at baseline, encompassing both actively cycling and persistently non-proliferative cellular states. Although the origin of these long-term non-dividing cells remains unclear, their occurrence raises the possibility that entry into proliferative arrest is influenced by lineage history rather than arising solely through stochastic state transitions. Such lineage-dependent behaviour would be consistent with non-genetic inheritance of cellular state across generations (Coller et al., 2006; Spencer et al., 2009; Shaffer et al., 2017; Emert et al., 2021; Shen et al., 2020). Future analyses of pedigree structure may reveal whether ancestral cellular states predispose specific lineages to enter persistent non-proliferative states.

The presence of a persistent non-dividing subpopulation within an otherwise dynamically heterogeneous baseline population is consistent with a bet-hedging framework, in which phenotypic diversification enables a fraction of cells to adopt dormant-like states that may enhance survival under future environmental stress. Rather than reflecting rare stochastic failures, these states may constitute an intrinsic component of adaptive population dynamics in clonal cancer cell systems (Oren et al., 2021; Goyal et al., 2023; Iyer et al., 2025; Hayford et al., 2026). To support this interpretation, growing evidence suggests that persister-like cells can arise from actively proliferating cells prior to treatment, rather than exclusively from pre-existing quiescent subpopulations (Oren et al., 2021). Recent studies have also identified “memory genes” that may contribute to the inheritance and or maintenance of these pre-existing cellular states (Shaffer et al., 2020; Emert et al., 2021; Ghosh et al., 2025). Such non-genetic memory provides potential mechanisms by which reversible phenotypic transitions can nevertheless produce lineage dependent patterns of behaviour.

Our findings are also consistent with studies that demonstrate that cancer cells undergo reversible transitions between distinct phenotypic states through transient epigenetic regulation and stochastic fluctuations (Gupta et al., 2011; Sharma et al., 2010; Liau et al., 2017; Guler et al., 2017; Tirosh, 2022). Consistent with this framework, the lineage-resolved trajectories observed here, together with previous single-cell tracking studies, reveal extensive dynamic heterogeneity between cancer cell lineages (Korsnes and Korsnes, 2015; 2018; 2023; Quinsgaard et al., 2024; Korsnes et al., 2024).

The challenge of resolving outliers in untreated populations is compounded by the limitations of conventional bulk and endpoint-based assays, which cannot capture continuous single-cell dynamics. In this context, 72-h single-cell lineage tracking provides a non-invasive framework to resolve temporal trajectories at cellular resolution, enabling direct observation of state transitions without perturbation. This differs fundamentally from retrospective barcoding approaches, which infer pre-existing heterogeneity from endpoint distributions (Oren et al., 2021) but do not resolve dynamic behavioural trajectories. Similarly, while time-lapse imaging studies have demonstrated spontaneous and transient entry into quiescent states associated with endogenous replication stress (Arora et al., 2017; Pulianmackal et al., 2021), continuous lineage reconstruction allows these events to be placed within a full genealogical and temporal context (Hilsenbeck et al., 2016; Chakrabarti et al., 2018; Ulicna et al., 2021).

Figure 6 shows the empirical joint distribution of lifetimes for sister cells of generations I and II. Lifetimes for sister cells are clearly positively correlated, suggesting inheritance. The figure confirms the finding of Figure 5 that cells tend to postpone their division at the start of video recording. The distribution for generation II exhibits greater variation. This suggests that cells experience stress at the start of recording, proving that video tracking experiments should cover several cell divisions. Figure 6 also shows correlation of intermitotic times for sister cells. Different types of inheritance can explain this observation.

Intercellular interactions can trigger coordinated behaviours resembling flocking, potentially introducing substantial variability between experiments that might otherwise appear identical (Spencer et al., 2009; Stuart and Satija, 2019; Sharma et al., 2010; Shen et al., 2025). This collective behaviour may explain the differences illustrated in Figures 8–11. Alternatively, the observed differences may reflect traits inherited from a small number of ancestral cells that influence subsequent cell division. Horizontal exchange of genetic or cytoplasmic material between lineages could weaken these lineage-dependent effects. Consistent with this possibility, the present experiments recorded 295 cell fusion events (Table 2), and lineage tracking showed that some re-fusions and fused cells subsequently underwent multipolar divisions, directly linking these events to abnormal mitosis.

Additional mechanisms of inter-lineage exchange may include the uptake of apoptotic bodies, extracellular DNA released from dying cells, and biomolecules transferred via extracellular vesicles. These observations raise the possibility that apoptosis can contribute to the evolution of cancer cell populations by facilitating the horizontal transfer of biological material between lineages. Single-cell video tracking provides a direct means of observing such processes and may help elucidate the mechanisms underlying tumour evolution. In the longer term, improved understanding of these dynamic cellular interactions may contribute to the development of more specific prognostic and therapeutic strategies, bridging *in vitro* observations with *in vivo* cancer biology.

## 5 Concluding remarks

Characterising untreated cell populations is essential for understanding the intrinsic sources of non-genetic heterogeneity that may influence experimental reproducibility and therapeutic responses. Conventional *in vitro* assays typically focus on drug-treated cells, implicitly assuming that untreated controls represent a stable and homogeneous baseline. Our findings challenge this assumption by demonstrating substantial phenotypic diversity already present before therapeutic perturbation.

The proposed approach is simple, reproducible and based on the tracking of a widely available cell line under standard culture conditions. Inter-mitotic time provides an objective proxy for proliferative behaviour and could potentially be incorporated into automated microscope software for routine quality control and early detection of unexpected cellular dynamics.

Analysis of 4,000 untreated MDA-MB-468 cell lineages revealed extensive baseline heterogeneity. Only 22% of the initial cells generated healthy pedigree trees, while most lineages exhibited prolonged cell cycles, cell death, abnormal divisions, or fusion events. In addition, a small but distinct population of cells remained non-dividing throughout the 72-h observation period, suggesting the presence of stable quiescent- or senescence-like states even without external perturbation.

These observations indicate that non-genetic heterogeneity and sensitivity to small perturbations are intrinsic properties of untreated cancer cell populations. Such variability is unlikely to be eliminated entirely by improved experimental control and, instead, requires statistical characterisation. The coexistence of actively proliferating and persistently non-dividing cells is consistent with current models of phenotypic plasticity and bet-hedging, in which reversible cellular states enhance population adaptability. Our results therefore support the view that untreated cancer cell populations already contain dynamic behavioural diversity that can contribute to variability in subsequent treatment responses and motivate routine lineage-based characterisation of baseline cell populations before therapeutic intervention.

## Data Availability

The data analyzed in this study is subject to the following licenses/restrictions: The datasets used in the current study are available from the corresponding author upon reasonable request. Requests to access these datasets should be directed to monica.s.korsnes@ntnu.no.

## Footnotes

1
https://www.korsnesbiocomputing.no/
